# A review of crack growth models for near-neutral pH stress corrosion cracking on oil and gas pipelines

**DOI:** 10.1186/s43065-021-00042-1

**Published:** 2021-10-24

**Authors:** Haotian Sun, Wenxing Zhou, Jidong Kang

**Affiliations:** 1 Department of Civil and Environmental Engineering, The University of Western Ontario, London, ON N6A 5B9 Canada; 2 CanmetMATERIALS, Natural Resources Canada, Hamilton, ON L8P 0A5 Canada

**Keywords:** Buried pipeline, NNpHSCC, Crack growth model, Corrosion fatigue, Hydrogen embrittlement, Predictive accuracy

## Abstract

This paper presents a review of four existing growth models for near-neutral pH stress corrosion cracking (NNpHSCC) defects on buried oil and gas pipelines: Chen et al.’s model, two models developed at the Southwest Research Institute (SwRI) and Xing et al.’s model. All four models consider corrosion fatigue enhanced by hydrogen embrittlement as the main growth mechanism for NNpHSCC. The predictive accuracy of these growth models is investigated based on 39 crack growth rates obtained from full-scale tests conducted at the CanmetMATERIALS of Natural Resources Canada of pipe specimens that are in contact with NNpH soils and subjected to cyclic internal pressures. The comparison of the observed and predicted crack growth rates indicates that the hydrogen-enhanced decohesion (HEDE) component of Xing et al.’s model leads to on average reasonably accurate predictions with the corresponding mean and coefficient of variation (COV) of the observed-to-predicted ratios being 1.06 and 61.2%, respectively. The predictive accuracy of the other three models are markedly poorer. The analysis results suggest that further research is needed to improve existing growth models or develop new growth models to facilitate the pipeline integrity management practice with respect to NNpHSCC.

## Introduction

Steel oil and gas pipelines are part of critical infrastructure systems in a modern society. There are about 4,000,000 and 840,000 km of transmission, gathering, feeder, and distribution pipelines in the US and Canada [1, 2], respectively, most of which are buried underground. The structural integrity of pipelines is threatened by various failure mechanisms such as the third-party interference, corrosion, stress corrosion cracking and ground movement. Failures of pipelines can have severe safety, environmental and economic consequences. The present study focuses on one of the leading causes of failure for buried pipelines [3–9], namely the near-neutral pH stress corrosion cracking (NNpHSCC). NNpHSCC defects on pipelines grow over time and compromise the pipeline’s pressure containment capacity, i.e. burst capacity. If unmitigated, such defects may lead to significant failure incidents such as the rupture and subsequent fire on a 914 mm-diameter natural gas pipeline near Prince George, BC, Canada on October 9, 2018, and the rupture of a 609 mm-diameter natural gas pipeline near Unityville, PA, USA on June 9, 2015. To evaluate the growth rate of NNpHSCC defects with a reasonable accuracy is critically important for the pipeline integrity management program as it allows integrity engineers to predict the deterioration of the burst capacity of the pipeline with confidence and carry out effective, timely mitigation actions, if necessary. The objective of this study is to review the current understanding of the mechanism of NNpHSCC on pipelines and several existing models in the literature to predict the growth of NNpHSCC defects, and to examine the accuracy of these growth models based on experimental data obtained from full-scale pipe specimens.

The rest of the paper is structured as follows. Section "NNpHSCC on pipelines" presents a review of the literature related to the mechanism of NNpHSCC on pipelines. Section "Growth models for NNpHSCC defects on pipelines" describes several NNpHSCC growth models proposed in the literature. Section "Accuracy of NNpHSCC crack growth models" describes a test program on the growth of NNpHSCC defects in full-scale pipe specimens conducted by researchers at Natural Resources Canada. A comparison between the SCC growth rates observed in the test program and corresponding growth rates predicted by the growth models reviewed is also presented in this section. Conclusions are presented in the last section.

## NNpHSCC on pipelines

Stress corrosion cracking (SCC) is defined as one type of environmentally assisted cracking (EAC), which occurs under the synergistic effects of corrosion reactions and mechanical stress [10]. SCC requires three essential factors present simultaneously to initiate and propagate: the tensile stress (mechanical factor), susceptible material (metallurgical factor), and corrosive environment (electrochemical factor) [11]. Two types of SCC have been identified on pipelines based on the electrolyte in contact with the metal surface: the high pH SCC and near-neutral pH SCC. The high pH SCC was first documented in Louisiana, US in the mid 1960s [12], whereas NNpHSCC was first reported on Canadian pipelines in the mid 1980s [13, 14]. NNpHSCC is so named because the local electrolyte has a pH value between 5.5 and 7.5 [11]. The tensile stress essential to the occurrence of NNpHSCC is mainly caused by the high internal operating pressure of the pipeline [15]. Cracks caused by NNpHSCC move across the grains of the pipe steel and are therefore transgranular. In contrast, cracks caused by high pH SCC move along the grain boundaries and are therefore intergranular [3].

The underlying mechanisms of NNpHSCC on pipelines have not been conclusively established. Many researchers [16–18] suggest that NNpHSCC is driven by the synergistic effect of hydrogen embrittlement (HE), anodic dissolution (AD), and cyclic stress. Parkins et al. [19] first suggested that both the dissolution and hydrogen ingress into the steel are responsible for the crack growth in NNpH environments. Gu et al. [20] proposed a hydrogen-facilitated anodic dissolution mechanism for NNpHSCC. Lu et al. [21] reported that the synergistic effect due to the interaction of dissolved hydrogen and local stress field on the active dissolution is negligible and suggested that NNpHSCC is unlikely to be controlled by the hydrogen-facilitated anodic dissolution mechanism based on their thermodynamic analysis and experimental observations. Lu et al. [22] further suggested that the crack propagation in pipeline steels in contact with NNpH groundwater is dominated by the dissolved hydrogen concentration and less influenced by AD. Cheng and his co-investigators also suggested that hydrogen plays a critical role in NNpHSCC of pipeline steels through the HE mechanism [23–25]. A recent experiment [17] demonstrated that about one-tenth of the measured NNpHSCC growth rate is due to AD, which implies that HE plays a dominant role in the NNpHSCC growth. HE occurs when hydrogen atoms enter the lattice of the metal and reduce its ductility and toughness. The atomistic mechanism for HE has been under investigation for the past several decades [26]. The following three theories of HE are widely referenced in the literature: 1) hydrogen-enhanced decohesion (HEDE), which postulates that hydrogen atoms trapped near a crack reduces the free surface energy, thus facilitating cleavage-like failure [27, 28]; 2) hydrogen-enhanced localized plasticity (HELP), which suggests that solute hydrogen enhances dislocation movements [29], and 3) adsorption-induced dislocation emission (AIDE), which hypothesizes that the adsorption of hydrogen facilitates the dislocation nucleation [30, 31].

Many researchers have claimed that cyclic stress is essential to the growth of NNpHSCC cracks [32–36]. Full-scale experiments showed that the absence of cyclic components in the loading spectra led to non-growth of NNpHSCC cracks in pipe specimens and that controlling pressure fluctuations in pipelines resulted in reduced crack growth [32]. A similar phenomenon was also observed in small-scale tests: no crack growth was detected in the specimen subjected to a monotonic loading even at the highest stress intensity factor used in the study [33]. It is therefore suggested in [34–36] that the growth of NNpHSCC cracks can be better characterized by corrosion fatigue (CF) than SCC. CF is one type of EAC that occurs under the synergistic effects of corrosion and cyclic stress [11, 37]. Note that internal pressures in pipelines are generally fluctuating, resulting in cyclic stresses in the pipeline. Although small-scale tests reported in [38–41] have shown that cracks can initiate and propagate in specimens in an NNpH environment under quasi-static and static loading conditions, extensive full-scale tests [42, 43] have demonstrated that cyclic stress facilitates the propagation of NNpH cracks far more than static stress. The present study considers CF enhanced by HE as the main mechanism for the growth of NNpH cracks. However, the terminology NNpHSCC is still adopted in the following discussions to be consistent with the literature and avoid confusion.

## Growth models for NNpHSCC defects on pipelines

### Growth models developed at SwRI

Chen and Sutherby [34] investigated the crack growth behaviour of the X65 pipe steel in NNpH environments by using compact tension (C(T)) specimens subjected to cyclic loads. They observed that the combined parameter, *K*_*max*_Δ*K*^2^*f*^-0.1^, results in the best fit to the experimentally-obtained d*a*/d*N* values corresponding to different stress ratios and loading frequencies. In the above, *a* denotes the crack depth (i.e. in the through pipe wall thickness direction); *N* denotes the number of stress cycles; d*a*/d*N* is the crack depth growth rate per stress cycle; *K*_*max*_ and Δ*K* are respectively the maximum stress intensity factor and stress intensity factor range in a load cycle, and *f* is the loading frequency. Lu [16] suggested that the term *K*_*max*_Δ*K*^2^ can be considered the mechanical parameter controlling the initiation of microcracks in the fracture process zone (FPZ) ahead of the crack tip and that the term *f*^-0.1^ represents the enhanced crack growth by the corrosive environment, whose effects decrease as *f* increases.

Based on Chen and Sutherby’s combined parameter, researchers at the Southwest Research Institute (SwRI) [44, 45] proposed the following crack growth model for NNpHSCC (referred to as the SwRI model):

1 $$\frac{da}{dN}={B}_0{\left[\ln \left(\frac{C_{cr}^{Lat}}{C_0}\right)\right]}^{-2}{\left({K}_{max}\Delta {K}^2{f}^{-0.1}\right)}^2$$

where *B*_0_ is a fitting coefficient; *C*_0_ is the atomic hydrogen concentration in the lattice of bulk steel, and *C*_*cr*_^*Lat*^ is the critical hydrogen concentration in the lattice of FPZ to cause the initiation of microcracks. The two main sources for the hydrogen consumed in the HE process are the hydrogen evolution reaction in the crack and hydrogen dissolved in the bulk steel [16, 46]. An experimental study carried out by Chen et al. [47] suggests that the latter is the primary source; therefore, *C*_0_ in Eq. (1) is replaced by *C*_*B*_, the hydrogen concentration in the bulk material, which can be determined from hydrogen permeation measurements [48]. The value of *C*_*B*_ is generally in the order of 10^− 2^ to 10^1^ mol/m^3^ depending on the pipe steel grade (representing the effect of the microstructure of steel), solution pH and steel potential [48]. Song et al. [44] developed the following empirical equation to estimate *C*_*B*_ as a function of the solution pH and steel potential (ignoring the influence of the steel grade):

2 $${C}_B={X}_{\mathrm{pH}}\frac{-\left(5+10\varphi \right){10}^{-10}\exp \left(-\frac{\upvarphi}{0.03}\right)}{-\left(5+10\varphi \right)+{10}^{-10}\exp \left(-\frac{\upvarphi}{0.03}\right)}\left(\mathrm{mol}/\mathrm{m}3\right)$$

3 $${X}_{\mathrm{pH}}=5-0.019{\left({10}^{6.3-\mathrm{pH}}-15.5\right)}^2\ \left(\mathrm{mol}/\mathrm{m}3\right)$$

where *φ* is the potential measured versus the copper/copper-sulfate reference electrode (CSE). Based on fitting to the experimental data, Song et al. [44] estimated the value of *C*_*cr*_^*Lat*^ and *B*_0_ in Eq. (1) to be 3.3 × 10^4^ mol/m^3^ and 1.9 × 10^− 13^ MPa^− 6^ m^− 2^ s^-1/5^, respectively. These parameters are obtained by linearly fitting three data points, each representing one type of NNpH solution and having one *C*_0_ value.

Lu [16] suggested mechanistic meanings of the SwRI model by proposing four basic hypotheses underpinning the model.

1. The crack propagation is dominated by CF enhanced by HE, and anodic dissolution effects are negligible.
2. The cracked body is at the steady state and under the small-scale yield condition.
3. The crack grows discontinuously through the mechanism of microcracks forming and developing in FPZ and eventually merging into the main crack.
4. The crack growth rate is approximately proportional to the size of FPZ.

Based on the above hypotheses, Lu [16] suggested that the interval in a loading cycle provides the time necessary for hydrogen to diffuse into FPZ; therefore, d*a*/d*N* is a function of the loading frequency *f* as reflected in Eq. (1). The crack growth rate increases as *f* decreases because longer time is available in a given load cycle for the hydrogen transport (diffusion). If *f* is below a lower threshold, all the microcracks in FPZ can connect with the main crack in one load cycle effect. In this case, the effect of *f* on the crack growth rate becomes saturated, and the growth rate is independent of *f*. If *f* is above an upper threshold, hydrogen atoms have insufficient time to diffuse to FPZ and participate in the crack growth process. In this case, d*a*/d*N* is dominated by the fatigue mechanism and independent of *f*.

A slightly modified SwRI model was further proposed by Lu [16] as follows:

4 $$\frac{da}{dN}={B}_0^{\prime }{\left[\ln \left(\frac{C_{cr}^{Lat}}{C_B}\right)\right]}^{-2}{\left(\frac{\Delta {K}_{eq}}{f^{\frac{1}{24}}}\right)}^6\ \mathrm{for}\left(\frac{\Delta {K}_{eq}}{f^{\frac{1}{24}}}\right)\ge {\left(\frac{\Delta {K}_{eq}}{f^{\frac{1}{24}}}\right)}_{th}$$

where Δ*K*_*eq*_ = *K*1/3 *max*Δ*K*^2/3^, *B*′ 0 = 8.8 × 10^− 14^ MPa^− 6^ m^−2^ s^-0.25^, and (Δ*K*_*eq*_/*f*^1/24^)_*th*_ is the threshold value for the combined parameter (Δ*K*_*eq*_/*f*^1/24^) below which the crack growth is considered negligible. Note that Lu [16] did not indicate the specific value of (Δ*K*_*eq*_/*f*^1/24^)_*th*_ or how it can be estimated. A comparison of Eqs. (1) and (4) reveals that the modified SwRI model differs slightly from the SwRI model in terms of the exponent on *f* on the right-hand side of the two equations: it is − 0.2 in Eq. (1) and − 0.25 in Eq. (4). Note that the latter value is obtained by fitting to the experimental data obtained in simulated groundwater with near-neutral pH [46, 49].

The development of the SwRI and modified SwRI models involves expressing the maximum hydrostatic stress in FPZ in terms of the stress intensity factor based on linear elastic fracture mechanics solutions for the crack-tip stress field. This, however, is problematic given that such solutions are inapplicable to the stress field within FPZ, which are associated with large strains and considerable plastic deformations.

### Xing et al.’s model

Xing et al. [50] proposed a growth model for NNpHSCC by considering both AIDE and HEDE, that is,

5 $$\frac{da}{dN}={\left(\frac{da}{dN}\right)}_{\mathrm{AIDE}+\mathrm{HEDE}}$$

**Fig. 1:**
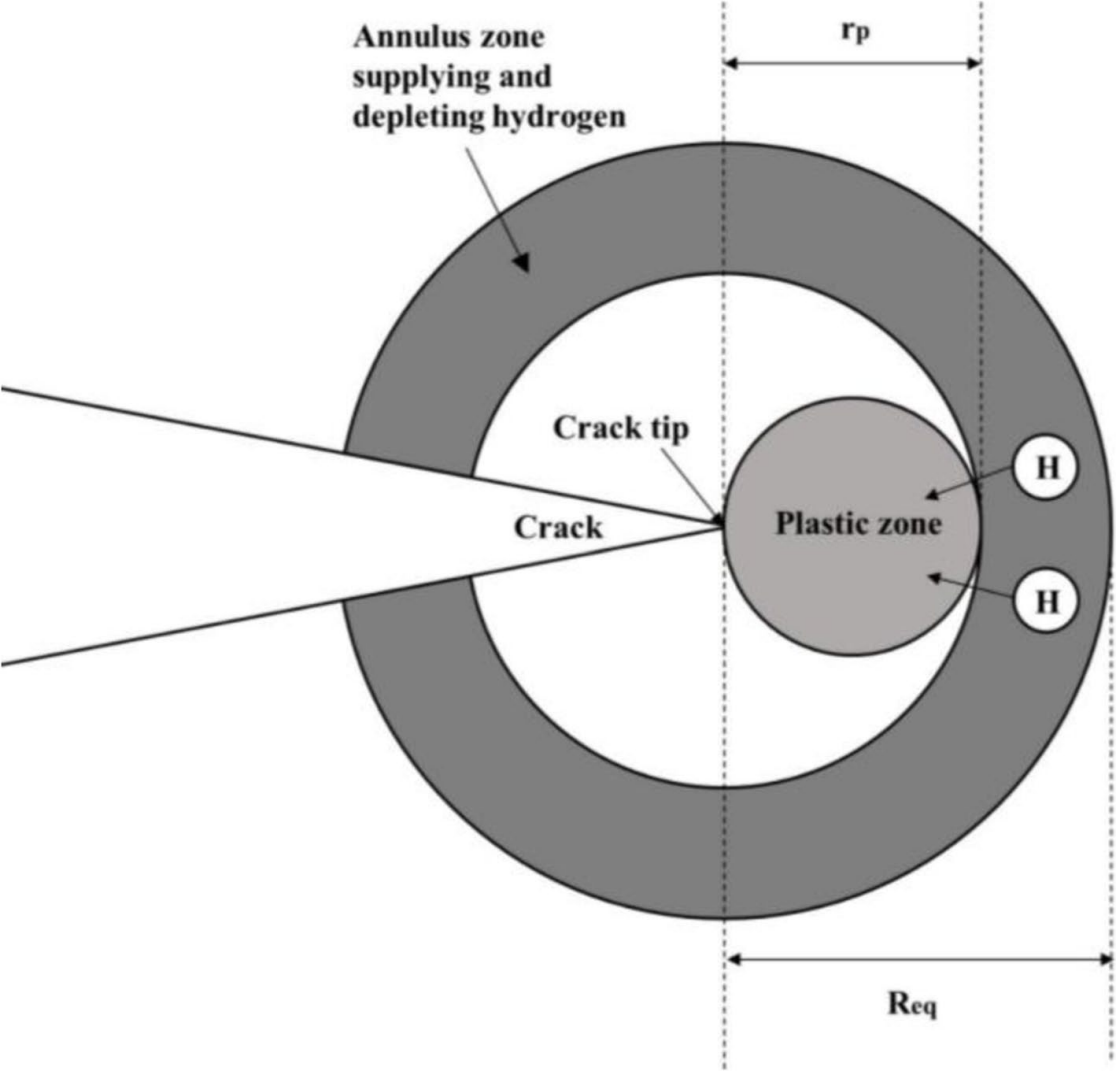
The schematic of the hydrogen enhanced crack growth model [50]

The crack growth rate due to HEDE only, (d*a*/d*N*)_HEDE_, considers the effects of hydrogen potential, diffusivity, hydrostatic stress near the crack tip and critical loading frequency [51–53], and is given by:

6 $${\left(\frac{da}{dN}\right)}_{\mathrm{HEDE}}=\left\{\begin{array}{c}{\left[\frac{4\left(1+\nu \right)\varOmega }{3\pi {k}_BT\sqrt{2\pi}\ln \left(\frac{1}{c_0}\right)}\right]}^2\frac{\left(\frac{1+R}{1-R}\right)\varDelta {K}^2}{{\left(\frac{f}{f_{crit}}\right)}^{\gamma }},f>{f}_{crit}\\ {}{\left[\frac{4\left(1+\nu \right)\varOmega }{3\pi {k}_BT\sqrt{2\pi}\ln \left(\frac{1}{c_0}\right)}\right]}^2\left(\frac{1+R}{1-R}\right)\varDelta {K}^2,f\le {f}_{crit}\end{array}\right.$$

7 $${f}_{crit}=\frac{\left(1+\nu \right)\varOmega D\left({K}_{max}+{K}_{min}\right)\left(\frac{1}{\sqrt{r_p}}-\frac{1}{\sqrt{R_{eq}}}\right)}{\pi {\left({R}_{eq}-{r}_p\right)}^2{k}_BT3\sqrt{2\pi }}$$

where *f*_*crit*_ represents the minimum loading frequency under which the crack growth rate reaches the maximum value and is independent of *f*; *ν* is Poisson’s ratio; Ω (m^3^) is the partial volume of hydrogen atom; *k*_*B*_ is the Boltzmann constant (= 1.3806 × 10^− 23^ m^2^ kg s^− 2^ K^− 1^); *T* (K) is the temperature; *c*_0_ is the atomic ratio of H/Fe away from the crack tip, which can vary from zero up to 5 × 10^− 4^ [50]; *R* = *K*_*min*_/*K*_*max*_ is the stress ratio; *γ* is a material constant to be obtained from data fitting; *D* is the hydrogen diffusivity rate (m^2^/s); *r*_*p*_ is the size of the plastic zone ahead of the crack tip (Fig. 1), and *R*_*eq*_ is the outer radius of the annulus region that supplies as well as depletes hydrogen atoms to the plastic zone during cyclic loading.

Xing et al. [50] further suggested that d*a*/d*N* can be related to (d*a*/d*N*)_HEDE_ through an empirical relationship: log(d*a*/d*N*)/log(d*a*/d*N*)_HEDE_ = *n*, where *n* is a fitted constant for a given steel. It follows from Eq. (6) that d*a*/d*N* is given by

8 $$\frac{da}{dN}=\left\{\begin{array}{c}{\left[\frac{4\left(1+\nu \right)\varOmega }{3\pi {k}_BT\sqrt{2\pi}\ln \left(\frac{1}{c_0}\right)}\right]}^{2n}{\left(\frac{\left(\frac{1+R}{1-R}\right)\varDelta {K}^2}{{\left(\frac{f}{f_{crit}}\right)}^{\gamma }}\right)}^n,f>{f}_{crit}\\ {}{\left[\frac{4\left(1+\nu \right)\varOmega }{3\pi {k}_BT\sqrt{2\pi}\ln \left(\frac{1}{c_0}\right)}\right]}^{2n}{\left(\left(\frac{1+R}{1-R}\right)\varDelta {K}^2\right)}^n,f\le {f}_{crit}\end{array}\right.$$

Based on fitting to the experimental data of the X65 and X52 steels [34, 54, 55], Xing et al. [28] recommended that *γ* be taken as 0.1 and *n* be taken as 0.92 and 0.88 for the X65 and X52 steels, respectively. It follows that the combined parameter in Xing et al.’s model is [(1 *+ R*)/(1*-R*)]Δ*K*^2^(*f*/*f*_*crit*_)^-0.1^, which is somewhat similar to the combined parameter, *K*_*max*_Δ*K*^2^*f*^-0.1^, proposed by Chen and Sutherby [34]. Xing et al. [56] suggested that the value of the combined parameter [(1 *+ R*)/(1*-R*)]Δ*K*^2^(*f*/*f*_*crit*_)^-0.1^ can be used to divide the crack growth process into three phases. A crack is in the dormant, initiation and fast growth phases if the corresponding value of [(1 *+ R*)/(1*-R*)]Δ*K*^2^(*f*/*f*_*crit*_)^-0.1^ is less than 500 MPa^2^ m, between 500 and 1000 MPa^2^ m, and greater than 1000 MPa^2^ m, respectively.

Xing et al. [50] did not recommend specific values of parameters Ω and *c*_0_ in Eq. (6). Bockris et al. [57] suggested the partial volume of hydrogen, *V*_*H*_, equals to 2.60 cm^3^/mol and 1.84 cm^3^/mol in α-Fe and AISI 4340 steel, respectively, at 27 °C under tensile stress, which is equivalent to 4.317 × 10^− 30^ m^3^ and 3.055 × 10^− 30^ m^3^ for the value of Ω, respectively. Lee and Gangloff [58] suggested *V*_*H*_ to equal 2.0 cm^3^/mol (Ω = 3.321 × 10^− 30^ m^3^) for ultra-high-strength steel. Yu et al. [59] suggested Ω to be 2.0 × 10^− 30^ m^3^, whereas Song and Curtin [52, 53] suggested Ω to equal 3.818 × 10^− 30^ m^3^. Song and Curtin [52] further suggested values of *c*_0_ for pipe steels of different grades: *c*_0_ equals 0.16 × 10^− 6^ and 0.12 × 10^− 6^ for the X52 and X42 steels, respectively. Xing [60] suggested *c*_0_ to equal 2.0 × 10^− 6^ regardless of the steel grade. Song and Curtin [52] suggested *D* to equal 2.7 × 10^− 11^ m^2^/s for both X52 and X42 steels. Xing et al. [50] argued that the diffusivity of hydrogen in steels under tension can be much higher than that in steels under zero stress, and recommended *D* to equal 1.7 × 10^− 9^ m^2^/s regardless of the steel grade. Yu et al. [59] further suggested that *D* could range from 1.5 × 10^− 9^ m^2^/s and 2.0 × 10^− 9^ m^2^/s with varying stress and strain.

### Chen et al.’s model

A modification of Xing et al.’s model was proposed by Chen and his co-investigators [61, 62] as follows:

9 $$\frac{da}{dN}={\left[\frac{4\sqrt{2.476}\left(1+\nu \right)\varOmega }{3\pi {k}_BT\sqrt{2\pi}\ln \left(\frac{1}{c_0}\right)}\right]}^{2{n}^{\prime }}{\left(\frac{\varDelta {K}^2{K}_{max}}{f^{0.1}}\right)}^{0.6{n}^{\prime }},f>{f}_{crit}$$

where *n’* = 2, and *f*_*crit*_ is suggested to be 10^− 3^ Hz. It is however unclear how the value of *n’* is estimated. All the other parameters in Eq. (9) have been defined previously. Chen et al.’s model differs from Xing et al.’s model in that the former employs the combined parameter proposed by Chen and Sutherby [34], i.e. *K*_*max*_Δ*K*^2^*f*^-0.1^. A few observations of Chen et al.’s model are noteworthy. First, the applicability of the model for *f* ≤ *f*_*crit*_ is not explicitly indicated by Chen et al., although it can be assumed that d*a*/d*N* is independent of *f* for *f* ≤ *f*_*crit*_ with d*a*/d*N* values for *f* ≤ *f*_*crit*_ equal to that for *f* = *f*_*crit*_. Second, care must be taken to ensure the consistency in the units of both sides of Eq. (9) due to the fact that the combined parameter in Eq. (9) involves the frequency directly, as opposed to a normalized frequency, i.e. *f*/*f*_*crit*_, employed in Xing et al.’s model. A dimensional analysis shows that the constant $\sqrt{2.476}$ on the right side of Eq. (9) must have a unit of m^-0.3^ s^-0.23^ kg^0.1^ to be compatible with the unit of m/cycle of d*a*/d*N*. This unit consistency requirement hinders the practical application of Chen et al.’s model.

## Accuracy of NNpHSCC crack growth models

### Crack growth data from tests of full-scale pipe specimens

Between 1993 and 1996, researchers at the CanmetMATERIALS (formerly Canmet Materials Technology Lab, or MTL) of Natural Resources Canada conducted full-scale NNpHSCC growth tests on three X52 pipes [32, 63]. The growth data collected from two of the three specimens (pipes #1 and #2) are employed in this study to validate the growth models described in Section 3; the other specimen is not considered because of limitations in the data recorded during the test. The outside diameters (*d*), wall thicknesses (*t*) of pipes #1 and #2 are 610 mm and 6.4 mm, respectively. The yield and tensile strengths (*σ*_*y*_ and *σ*_*u*_) determined from tensile coupon tests for the specimens are 421 and 538 MPa, respectively.

**Fig. 2:**
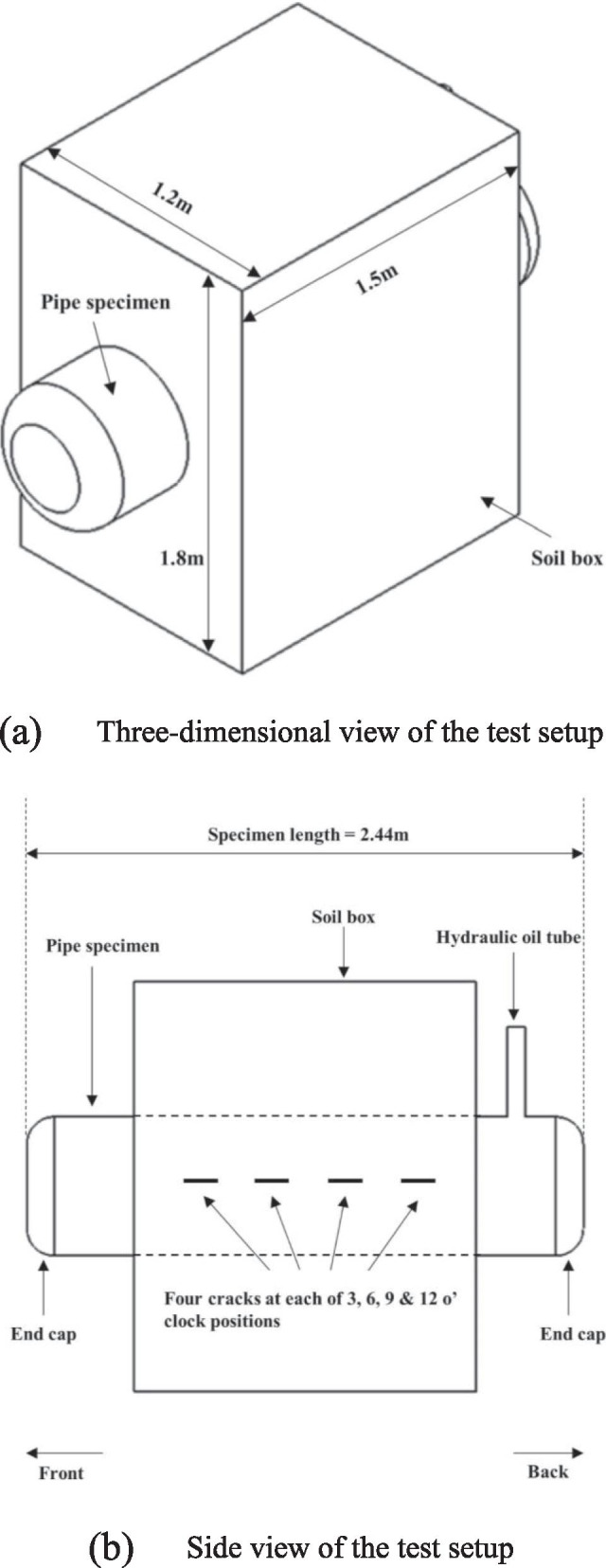
Schematic illustration of the test setup for the SCC growth test. **a** Three-dimensional view of the test setup. **b** Side view of the test setup

**Fig. 3:**
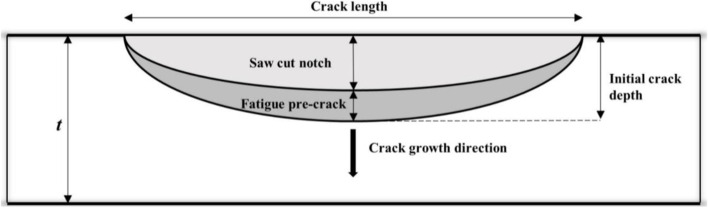
Schematic diagram of notch and pre-crack on pipe surface

Sixteen cracks equally distributed over four circumferential positions (i.e. 3, 6, 9 and 12 o’clock positions) were introduced on each of the two specimens. At a given clock position, four cracks are oriented along the longitudinal axis of the specimen with an end-to-end separation distance (*s*) of about 100 mm (Fig. 2). The specimens were internally pressurized by hydraulic oil. The pressure level and rate of loading were controlled by a feedback system consisting of a pressure gauge, servovalves, servovalve controllers and interfacing hardware. Two end plates were welded on each specimen to contain the internal pressure. The local NNpH environments for the cracks were realized by using a soil box enclosing the pipe external surface (Fig. 2). The soil box was filled with a clay-type soil collected from a failure site of a pipeline caused by NNpH SCC. The average pH of the soil environment around the pipe surface was maintained between 6.9 and 7.2 during the test. The initial depth of a crack is the sum of the depths of a saw-cut notch and the subsequent fatigue pre-crack (Fig. 3). The crack length (i.e. in the pipe axial direction) was made far greater than the depth during saw cutting to ensure that the crack propagates primarily in the depth direction with negligible length growth. According to BS7910 [64], two coplanar surface flaws with *a*_1_/*l*_1_ ≤ 1 and *a*_2_/*l*_2_ ≤ 1 are considered to interact if *s* ≤ max{*a*_1_/2, *a*_2_/2}, where *a*_1_ and *a*_2_ are depths of the two cracks, respectively, and *l*_1_ and *l*_2_ are half-lengths of the cracks, respectively. Since *s* for the cracks considered in the present study is far greater than the maximum crack depths (ranging from 2 to 5 mm in general), the cracks at the same clock position do not interact with each other. The initial crack depth was measured using a direct current potential drop (DCPD) system, which has a resolution of about 30 μm.

**Fig. 4:**
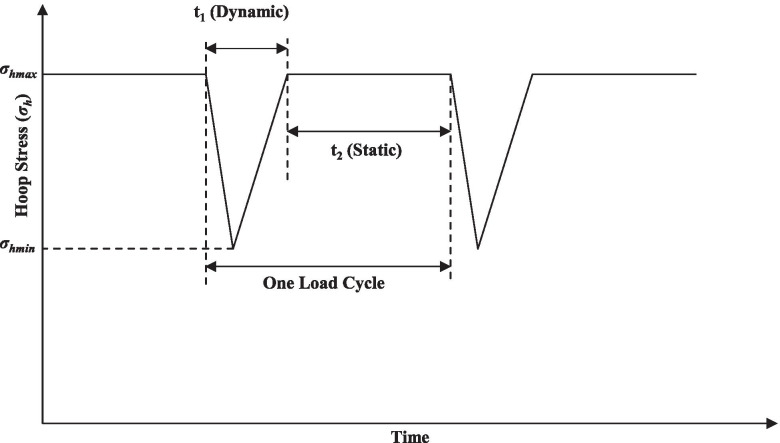
Stress cycle applied within a given test period for a pipe specimen

Each specimen was subjected to a series of different load spectra, referred to as the “test periods”, over the duration of the test. Fig. 4 illustrates the stress cycle applied within a given test period, which consists of a saw-tooth-shaped dynamic component and a static component. The unloading rate is twice the loading rate in the dynamic component. The inclusion of both the dynamic and static components in a stress cycle is intended to gain understanding of their respective effects on the crack growth rate. This can be achieved by comparing the crack growth rate obtained from the load spectrum illustrated in Fig. 4 with that corresponding to a reference load spectrum consisting only of the dynamic component in a stress cycle. The test data corresponding to such a reference load spectrum are, however, unavailable. During the test, the crack depth was measured by DCPD at different times such that the total crack growth (i.e. difference between the crack depth at the time of measurement and initial crack depth) was tracked throughout the test. Note that due to the uncertainties associated with the DCPD measurement as well as generally slow growths of cracks, the DCPD-measured crack depth did not monotonically increase with time.

Once the test was completed, the actual final depth of each crack on pipe #1 was physically measured by breaking open the pipe specimen at the location of the crack and compared with the corresponding final crack depth measured by DCPD to validate the accuracy of the DCPD measurement. Researchers at Canmet considered the DCPD-measured final depths of 9 cracks on pipe #1 to be close to the corresponding physically-measured crack depths, i.e. the measurement error of DCPD is considered acceptable. The final depths of the cracks on pipe #2 were not physically measured. The four cracks at the 9 o’clock position on pipe #2 are in the seam weld of the specimen and excluded in the present study because it is unclear if the growth models described in Section 3 are applicable to cracks in the weldment. It is assumed in this study that the DCPD-measured depths of the remaining 12 cracks on pipe #2 (i.e. at the 3, 6 and 12 o’clock positions) are associated with acceptable measurement errors. Therefore, a total of 21 cracks (9 from pipe #1 and 12 from pipe #2) are considered in the subsequent analysis to evaluate the accuracy of the crack growth models as detailed in Section 4.2. Table 1 summarizes the initial and final depths, and lengths of these 21 cracks. Tables 2 and 3 summarizes the relevant information of the test periods associated with the two pipe specimens, including the maximum hoop stress (*σ*_*hmax*_) within a given stress cycle, *R* (= *σ*_*hmin*_/*σ*_*hmax*_), durations of the dynamic and static components of one stress cycle (*t*_1_ and *t*_2_), and duration of each test period.

**Table 1:** Depths and lengths of 21 cracks used to evaluate the accuracy of the growth models

No.	Crack ID	Initial crack depth^b^ (mm)	Length (mm)	Final crack depth^b^ (mm)
1	1–3-1^a^	1.72	90	1.87
2	1–3-2	1.67	75	1.82
3	1–6-1	2.04	26.5	2.14
4	1–6-2	2.72	37.5	3.17
5	1–6-3	2.29	37	2.79
6	1–9-1	2.80	36	5.20
7	1–9-2	2.73	37	5.93
8	1–12-1	2.11	37	2.16
9	1–12-2	1.73	26	2.18
10	2–3-1	1.70	36	1.79
11	2–3-2	2.31	46	2.44
12	2–3-3	1.80	36	1.93
13	2–3-4	2.85	46	3.25
14	2–6-1	1.90	36	2.00
15	2–6-2	2.30	46	2.60
16	2–6-3	1.90	36	2.00
17	2–6-4	2.50	46	2.72
18	2–12-1	2.10	36	2.23
19	2–12-2	3.00	46	3.22
20	2–12-3	2.20	36	2.27
21	2–12-4	3.00	46	3.30

^a^The number (1 or 2) before the first hyphen in the crack ID indicates the specimen on which the crack is located; the number after the first hyphen (3, 6, 9 or 12) indicates the clock position of the crack; the number after the second hyphen (1, 2, 3 or 4) identifies the specific crack at that clock position

^b^Initial crack depths of cracks No. 1 to 9 are physically measured while initial crack depths of cracks No. 10 to 21 are DCPD measured; final crack depths are obtained by adding DCPD measured SCC growths to initial crack depths

**Table 2:** Information on the test periods for pipe#1

Test period	*σ*_*hmax*_/*σ*_*y*_	*R*	*t*_1_, *t*_2_ (min)	Duration (days)
I	0.55	0.80	20, 153	21
II	0.67	0.80	20, 153	19
III	0.72	0.82	20, 153	36
IV	0.75	0.80	10, 30	10
V	0.75	0.63	10, 30	32
VI	0.80	0.60	5, 10	38
VII	0.80	0.55	5, 10	39
VIII	0.80	0.90	5, 10	32
IX	0.77	0.80	20, 5	20

**Table 3:** Information on the test periods for pipe#2

Test period	*σ*_*hmax*_/*σ*_*y*_	*R*	*t*_1_, *t*_2_ (min)	Duration (days)
I	0.55	0.57	10, 30	60
II	0.67	0.53	10, 30	55
III	0.67	0.80	10, 30	30
IV	0.77	0.80	20, 5	60
V	0.77	0.80	5, 20	105

### Evaluation of crack growth rates based on test data

**Fig. 5:**
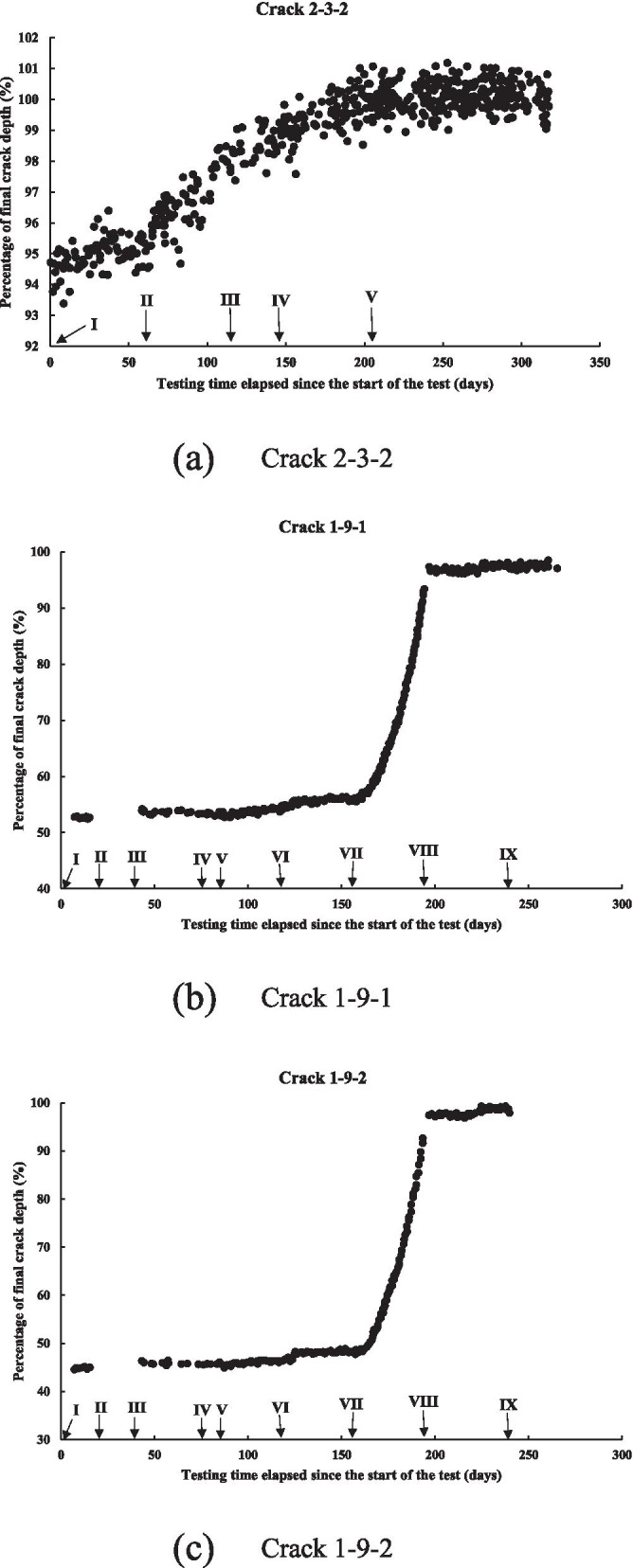
Crack growth over the test duration for cracks 2–3-2, 1–9-1 and 1–9-2 [63]. **a** Crack 2–3-2. **b** Crack 1–9-1. **c** Crack 1–9-2

All of the 21 cracks listed in Table 1 except cracks 1–9-1 and 1–9-2 grew slowly during the test. Fig. 5 depicts the crack growth over the duration of the test for a representative crack, 2–3-2, as well as cracks 1–9-1 and 1–9-2. Cracks 1–9-1 and 1–9-2 differ from the other 19 cracks in that they exhibited fast growths during the test: the final crack depths (5.20 and 5.93 mm) are approximately twice the initial crack depths (2.80 and 2.73 mm). Figure 5b and c reveal that the fast growths of cracks 1–9-1 and 1–9-2 during the test are due entirely to their growths in test period VII, which should be considered separately. On the other hand, cracks 1–9-1 and 1–9-2 grow slowly in all test periods other than VII. For each of the 21 cracks in Table 1, the crack growth rate is evaluated for a selected number of test periods during which the crack growth trend is reasonably clear from the DCPD measurements. Since it is very difficult to quantify the change in the crack growth rate of a given slowly-growing crack within a given test period due to the relatively large scatter in the DCPD measurements, a constant crack growth rate, d*a*/d*t* (mm/s), is evaluated for a given test period based on the linear regression analysis of the DCPD data. This results in a total of 39 d*a*/d*t* values for the 21 cracks within the selected test periods (see Table 7 of Appendix). These 39 growth rates are referred to as the dataset hereafter. The linear regression results corresponding to the d*a*/d*t* values are included in Figure 7 of Appendix. Note that the crack growth rates included in the dataset are generally in the order of 10^− 8^ mm/s (0.32 mm/year). This is consistent with typical growth rates of NNpHSCC observed on in-service pipelines [4].

Quadratic equations are found to fit very well the growth paths of cracks 1–9-1 and 1–9-2 within test period VII. It follows that multiple d*a*/d*t* values can be obtained for cracks 1–9-1 and 1–9-2 within test period VII by evaluating the slopes of the two fitted quadratic equations at different times. However, the obtained growth rates are generally in the order of 10^− 7^ – 10^− 6^ mm/s (3.2–32 mm/year), which are order-of-magnitude higher than typical NNpHSCC growth rates observed in practice. Therefore, these growth rates are not considered in the subsequent analysis.

### Model predicted crack growth rates

The four growth models described in Section "Growth models for NNpHSCC defects on pipelines", i.e. the SwRI and modified SwRI models, Xing et al.’s and Chen et al.’s models, are employed to predict the growth rates included in the dataset. Analysis results however revealed that the crack growth rates predicted by Chen et al.’s model are drastically different from the corresponding growth rates obtained in the test. Therefore, Chen et al.’s model is not discussed further in the following sections, and predictions by the other three growth models are described in detail. In applying the modified SwRI model, the threshold value (Δ*K*_*eq*_/*f*^1/24^)_*th*_ is not considered in the calculation since crack growths had been observed during all 39 collected test periods. In applying Xing et al.’s model, the value of *n* in Eq. (8) is set to 0.88 corresponding to the X52 pipe steel. Furthermore, the values of (d*a*/d*N*)_HEDE_ in Xing et al.’s model, i.e. Eq. (6), are also evaluated for the dataset. The crack growth rate per cycle, i.e. d*a*/d*N*, predicted by the growth model is converted to d*a*/d*t* using the following equation:

10 $$\frac{da}{dt}=f\frac{da}{dN}$$

where *f* is the frequency of the cyclic load, which is a constant within a given test period and equals 1/(*t*_1_ + *t*_2_) (Fig. 4). The values of *f* in various test periods for pipes #1 and #2 range from 9.6 × 10^− 5^ to 1.1 × 10^− 3^ Hz. It is noteworthy that the stress cycle applied to the pipe specimen consists of the dynamic and static components. Previous studies [32, 65] suggest that the static load does not cause the growth of NNpHSCC. Further studies are therefore needed to investigate if the stress cycle shown in Fig. 4 could be converted to an equivalent stress cycle that consists of the dynamic component only.

All three growth models involve the evaluation of the maximum stress intensity factor within a stress cycle (*K*_*max*_) to predict the crack growth rates. To this end, a given crack is assumed to have a semi-elliptical profile and grows in the depth direction only. The Raju-Newman equation [66] is then employed to evaluate the stress intensity factor at the deepest point of the crack front. Since each of the d*a*/d*t* values included in the dataset is considered the average observed crack growth rate of a given crack within a certain test period, the corresponding predicted d*a*/d*t* is evaluated by using a single *K*_*max*_ value in the crack growth model, which is the average of the *K*_*max*_ values corresponding to all the crack depth measurements included in the test period for the crack. This simplification is justified by the fact that the increase in *K*_*max*_ within a given test period is generally less than 5% for the cracks included in the dataset.

For clarity and easy reference, Table 4 summarizes values of parameters of the three growth models, i.e. *B*_0_, *B*′ 0, *CLat cr*, *C*_*B*_, *D*, *r*_*p*_, *R*_*eq*_, Ω and *c*_0_, adopted in the present study as well as the corresponding sources for the values.

**Table 4:** Model parameters employed in predictions

Model	Parameter	Value or equation	Source
SwRI	*B*_0_	1.9 × 10^−13^ MPa^−6^ m^−2^ s^-0.2^	[44]
Modified SwRI	${B}_0^{\prime }$	8.8 × 10^− 14^ MPa^− 6^ m^− 2^ s^-0.25^	[16]
SwRI & Modified SwRI	${C}_{cr}^{Lat}$	3.3 × 10^4^ mol/m^3^	[44]
SwRI & Modified SwRI	*C*_*B*_	0.447 mol/m^3^	Eqs. (2) and (3)^1^
Xing et al.’s	*D*	1.7 × 10^− 9^ m^2^/s	[50]
Xing et al.’s	*r*_*p*_	$\left(\frac{1}{6\pi}\right){\left(\frac{K_{max}}{\sigma_y}\right)}^2$	[50]
Xing et al.’s	*R*_*eq*_	(*r*_*p*_ + 0.13) mm	[59]
Xing et al.’s & Chen et al.’s	Ω	2.0 × 10^− 30^ m^3^	[59]
Xing et al.’s & Chen et al.’s	*c*_0_	0.16 × 10^− 6^	[52]

^1^The value of *C*_*B*_ is calculated using Eqs. (2) and (3) by assuming pH = 7 and φ  = − 0.7V_CSE_

### Comparison of observed and predicted crack growth rates

**Fig. 6:**
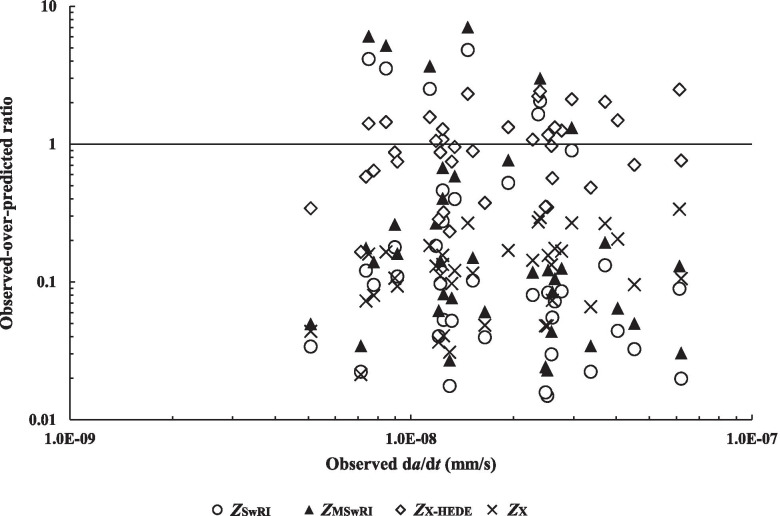
Comparison of observed and predicted crack growth rates for the dataset

**Fig. 7:**
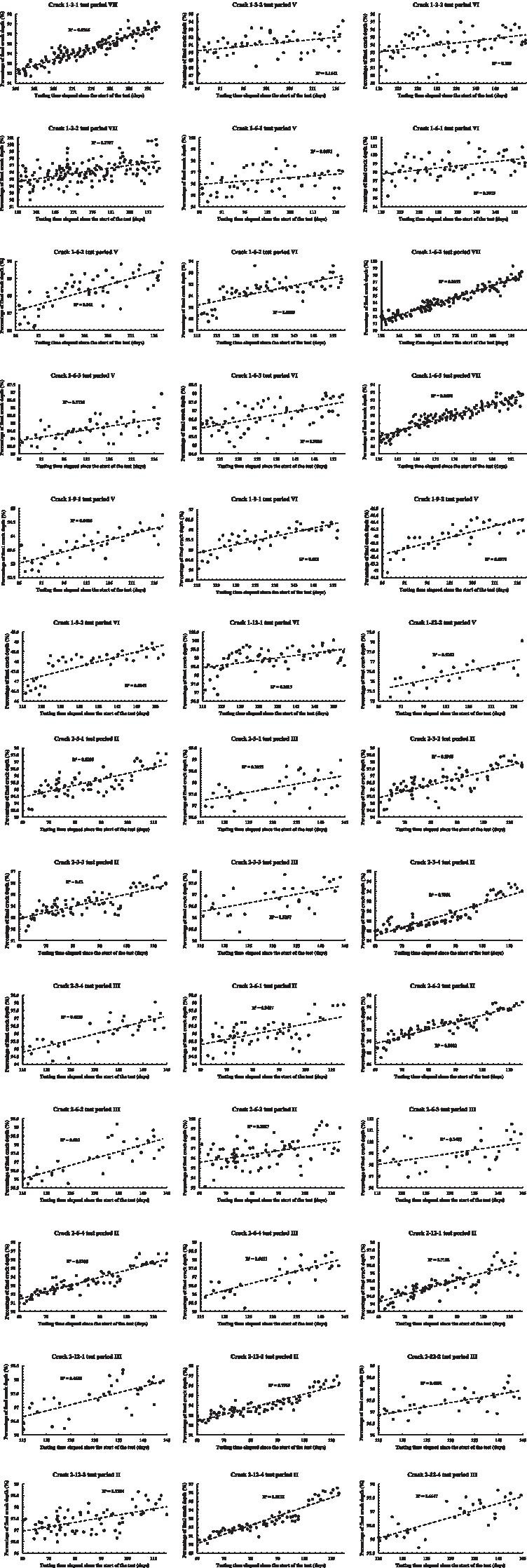
1 Linear regression results corresponding to the crack growth rates in the dataset

Predictions by the three growth models are shown in Fig. 6 for the dataset by plotting ratios of observed to predicted growth rates versus the observed growth rates. Let *Z*_SwRI_ and *Z*_MSwRI_ denote the observed-to-predicted growth rates corresponding to the SwRI and Modified SwRI models, respectively; let *Z*_X-HEDE_ and *Z*_X_ denote the observed-to-predicted growth rates corresponding to the HEDE component of Xing et al.’s model (Eq. (6)) and Xing et al.’s model (Eq. (8)), respectively. The mean and coefficient of variation (COV) of *Z*_SwRI_, *Z*_MSwRI_, *Z*_X_ and *Z*_X-HEDE_ for the dataset are shown in Table 5.

**Table 5:** Mean and COV of observed-to-predicted ratios for the growth models

	*Z*_SwRI_	*Z*_MSwRI_	*Z*_X-HEDE_	*Z*_X_
Mean	0.59	0.87	1.06	0.13
COV (%)	200.5	200.4	61.2	60.1
Min	0.01	0.02	0.16	0.02
Max	4.81	7.04	2.49	0.34

Table 5 indicates that the accuracy of the predicted growth rates varies widely. The HEDE component of Xing et al.’s model leads to the best predictions for the dataset, with the mean and COV of *Z*_X-HEDE_ equal to 1.06 and 61.2%, respectively. It is interesting to note that Xing et al.’s model performs much poorer than its HEDE component and results in on average almost one order of magnitude over-predictions of the growth rates in the dataset. Although the two SwRI models are on average somewhat accurate to predict the growth rates in the dataset, there is large variability associated with the predictions: the corresponding COVs are 200%, and the minimum and maximum observed-to-predicted ratios differ by two orders of magnitude. This can be explained by the strong dependence of the predicted growth rates on Δ*K*: for both the SwRI models, the predicted growth rates are proportional to Δ*K*^4^. An increase of *R* from 0.6 to 0.8 will lead to a 94% reduction in the predicted crack growth rate, all else being the same; however, such marked changes were not observed in the observed crack growth rates. These observations suggest that further research is needed to determine a more appropriate exponent on Δ*K* in both SwRI models.

While there is little room to adjust the value of a given parameter in both SwRI models as evident from the description in the previous Section, the possible value of a given parameter in Xing et al.’s model (e.g. *D*) may vary within a wide range. It is therefore valuable to investigate which input parameters have the most significant influences on the accuracy of Xing et al.’s model such that more efforts can be made to quantify those parameters more accurately. As the HEDE component of Xing et al.’s model leads to the best predictions for the dataset, analyses are carried out to investigate the sensitivity of *Z*_X-HEDE_ to the values of different parameters. To this end, three parameters, namely *D*, Ω and *c*_0_, are considered in the sensitivity analysis as they have largely different recommended values from different sources: *D* varies from 2.7 × 10^− 11^ m^2^/s to 2.0 × 10^− 9^ m^2^/s [50, 52, 59]; Ω varies from 2.0 × 10^− 30^ m^3^ to 4.317 × 10^− 30^ m^3^ [52, 53, 57–59], and *c*_0_ varies from 0 to 5 × 10^− 4^ as suggested in [50, 52, 60]. For each parameter, three values are considered in the sensitivity analysis: one base case corresponding to the value indicated in Table 4 and two sensitivity cases as summarized in Table 6. In the sensitivity cases corresponding to a given parameter, values of all the other parameters are kept the same as those listed in Table 4. It is worth noting the relationship between *f*_*crit*_ and *f* for the dataset of the growth rates. For the base case, the majority of the data points (33 out of 39) have *f* < *f*_*crit*_, indicating that the corresponding predicted growth rates are independent of *f*. Since *f*_*crit*_ is independent of *c*_0_, the two sensitivity cases for *c*_0_ are the same as the base case in terms of the relationship between *f*_*crit*_ and *f* for the dataset. On the other hand, *f*_*crit*_ is a function of *D* and Ω. The two sensitivity cases for *D* result in all 39 data points having *f* > *f*_*crit*_, whereas the two sensitivity cases for Ω result in all 39 data points having *f* < *f*_*crit*_. The results of the sensitivity analysis as summarized in Table 6 indicate that the COV of *Z*_X-HEDE_ is only marginally affected by varying values of *D*, Ω and *c*_0_, whereas the mean of *Z*_X-HEDE_ is somewhat influenced by varying values of *D*, Ω and *c*_0_. Overall, the base case values of *D*, Ω and *c*_0_, i.e. those listed in Table 4, result in relatively more accurate model predictions.

**Table 6:** Sensitivity analyses with respect to three parameters in the HEDE component of Xing et al.’s model

Parameter	Case	Value	*Z*_X-HEDE_		Remark
			Mean	COV (%)	
*D* (m^2^/s)	Base case	1.7 × 10^− 9^	1.06	61.2	
	Sensitivity #1	2.7 × 10^−11^	1.46	59.8	Value suggested in [52]
	Sensitivity #2	2.0 × 10^−10^	1.20	59.8	Intermediate value between base case and sensitivity #1
Ω (m^3^)	Base case	2.0 × 10^−30^	1.06	61.2	
	Sensitivity #1	3.818 × 10^−30^	0.29	61.6	Value suggested in [52, 53]
	Sensitivity #2	4.317 × 10^− 30^	0.23	61.6	Value suggested in [57]
*c*_0_	Base case	0.16 × 10^− 6^	1.06	61.2	
	Sensitivity #1	2 × 10^− 6^	0.74	61.2	Value suggested in [60]
	Sensitivity #2	5 × 10^− 4^	0.25	61.2	Value suggested in [50]

## Conclusions

This study presents a review of four existing growth models for NNpHSCC defects on buried oil and gas pipelines, namely the two models developed at SwRI, Xing et al.’s model and Chen et al.’s model. All four models assume the main growth mechanism for NNpHSCC defects to be corrosion fatigue enhanced by hydrogen embrittlement. To investigate the predictive accuracy of the four models, a dataset consisting of 39 crack growth rates is established from a test program involving full-scale pipe specimens in NNpH environment under cyclic internal pressures conducted at Natural Resources Canada. The crack growth rates in the dataset are in the order of 10^− 8^ mm/s (0.32 mm/year), consistent with typical NNpHSCC growth rates observed on in-service oil and gas pipelines. The growth rates of the 39 cracks in the dataset are predicted using each of the four growth models, and the predicted growth rates are then compared with the corresponding observed growth rates.

The analysis reveals that Chen et al.’s model results in highly inaccurate predictions of the observed growth rates, whereas the SwRI model, Modified SwRI model and Xing et al.’s model lead to on average reasonably accurate predictions. However, predictions by both SwRI models are associated with high variability, with the COV of the observed-to-predicted growth rates equal to 200%. The HEDE component of Xing et al.’s model leads to the best predictions with the mean and COV of the observed-to-predicted ratios equal to 1.06 and 61.2%, respectively. Analyses further indicate that the accuracy of the HEDE component of Xing et al.’s model is somewhat sensitive to the values of three model parameters (i.e. *D*, Ω and *c*_0_). The findings of this study suggest that further research is needed to improve the existing NNpHSCC growth models or develop new growth models such that adequately accurate predictions of the NNpHSCC growth rates can be achieved in the pipeline integrity management practice.

## Data Availability

The dataset of the crack growth rates employed in the present study are already included in the manuscript. The raw data from which the crack growth rates are obtained are unavailable due to the non-disclosure agreement between the University of Western Ontario and Natural Resources Canada.

## Appendix

### Details of crack growth rates obtained from full-scale tests

Figure 7

Table 7

**Table 7:** 1 Summary of 39 observed crack growth rates obtained from the full-scale tests

No.	Crack ID	Test period ID	Observed d*a*/d*t* (10^−8^ mm/s)
1	1–3-1	VII	2.6
2	1–3-2	V	1.2
3	1–3-2	VI	1.2
4	1–3-2	VII	1.6
5	1–6-1	V	0.7
6	1–6-1	VI	1.2
7	1–6-2	V	2.8
8	1–6-2	VI	2.5
9	1–6-2	VII	6.2
10	1–6-3	V	1.5
11	1–6-3	VI	1.3
12	1–6-3	VII	4.5
13	1–9-1	V	2.5
14	1–9-1	VI	2.5
15	1–9-2	V	2.3
16	1–9-2	VI	3.4
17	1–12-1	VI	0.7
18	1–12-2	V	1.2
19	2–3-1	II	0.9
20	2–3-1	III	0.8
21	2–3-2	II	1.3
22	2–3-3	II	1.2
23	2–3-3	III	0.9
24	2–3-4	II	6.1
25	2–3-4	III	3.0
26	2–6-1	II	0.8
27	2–6-2	II	3.7
28	2–6-2	III	2.4
29	2–6-3	II	0.9
30	2–6-3	III	1.5
31	2–6-4	II	2.6
32	2–6-4	III	2.4
33	2–12-1	II	1.2
34	2–12-1	III	1.1
35	2–12-2	II	2.6
36	2–12-2	III	1.3
37	2–12-3	II	0.5
38	2–12-4	II	4.0
39	2–12-4	III	1.9

## Abbreviations

AD: Anodic dissolution
AIDE: Adsorption-induced dislocation emission
Canmet: CanmetMATERIALS Lab
CF: Corrosion fatigue
COV: Coefficient of variation
DCPD: Direct current potential drop
FPZ: Fracture process zone
HE: Hydrogen embrittlement
HEDE: Hydrogen-enhanced decohesion
HELP: Hydrogen-enhanced localized plasticity
NNpHSCC: Near-neutral pH stress corrosion cracking
SCC: Stress corrosion cracking
SwRI: Southwest Research Institute
